# Making medical student course evaluations meaningful: implementation of an intensive course review protocol

**DOI:** 10.1186/s12909-015-0387-1

**Published:** 2015-06-04

**Authors:** Patrick Fleming, Olga Heath, Alan Goodridge, Vernon Curran

**Affiliations:** 1 Faculty of Medicine, Memorial University of Newfoundland, St. John’s, Canada; 2 Division of Dermatology, University of Toronto, Toronto, Canada; 3 Center for Collaborative Health Professional Education and Division of Community Health & Humanities, Faculty of Medicine, Memorial University of Newfoundland, St. John’s, Canada; 4 Department of Medicine, Faculty of Medicine, Memorial University of Newfoundland, St. John’s, Canada; 5 Center for Collaborative Health Professional Education and Faculty of Education, Memorial University of Newfoundland, St. John’s, Canada; 6 Centre for Collaborative Health Professional Education, Faculty of Medicine, Memorial University of Newfoundland, Room 2901, Health Sciences Centre, St. John’s, NL A1B 3V6 Canada

## Abstract

**Background:**

Ongoing course evaluation is a key component of quality improvement in higher education. The complexities associated with delivering high quality medical education programs involving multiple lecturers can make course and instructor evaluation challenging. We describe the implementation and evaluation of an “intensive course review protocol” in an undergraduate medical program

**Methods:**

We examined pre-clerkship courses from 2006 to 2011 - prior to and following protocol implementation. Our non-parametric analysis included Mann-Whitney U tests to compare the 2006/07 and 2010/11 academic years.

**Results:**

We included 30 courses in our analysis. In the 2006/07 academic year, 13/30 courses (43.3 %) did not meet the minimum benchmark and were put under intensive review. By 2010/11, only 3/30 courses (10.0 %) were still below the minimum benchmark. Compared to 2006/07, courses ratings in the 2010/11 year were significantly higher (p = 0.004). However, during the study period mean response rates fell from 76.5 % in 2006/07 to 49.7 % in 2010/11.

**Conclusion:**

These results suggest an intensive course review protocol can have a significant impact on pre-clerkship course ratings in an undergraduate medical program. Reductions in survey response rates represent an ongoing challenge in the interpretation of student feedback.

**Electronic supplementary material:**

The online version of this article (doi:10.1186/s12909-015-0387-1) contains supplementary material, which is available to authorized users.

## Background

Ongoing course evaluation is a key component of quality improvement in higher education and an accreditation requirement for Canadian medical schools [1, 2]. The literature recommends a comprehensive approach to evaluation that is directly linked to curricular development and modification [3]. However, there is no identified best practice for achieving this goal [4]. The complexities associated with delivering high quality medical education programs involving multiple lecturers can make course and instructor evaluation quite challenging [5].

The most common evaluation model in medical education involves student course evaluation questionnaires completed at the conclusion of a course [4, 6]. Previous research has demonstrated that students are discriminating judges of instructional effectiveness, providing ratings that are quite stable [6] and valid indicators of teaching effectiveness [7, 8].

The majority of medical schools in both Canada and the United States have course evaluation systems in place based on student-completed questionnaires [4]. However, there is very little in the literature to help guide medical schools in creating evaluation system frameworks to maintain the quality of their undergraduate medical education programs. In particular, it is unclear how medical schools use student feedback to enhance course curriculum and if such changes improve future curriculum delivery [9, 10].

### Educational environment

The Faculty of Medicine at Memorial University of Newfoundland in St. John’s, Canada has a four-year doctor of medicine (MD) program requiring a bachelor’s degree for entry. During the study period, 60-64 students were admitted each year of which 52-56 were residents in the provinces of Newfoundland & Labrador, New Brunswick, or Prince Edward Island in Canada.

The first two years of the undergraduate medicine program are referred to as “pre-clerkship”. At the time, courses in pre-clerkship involved a combination of didactic lectures, case-based learning, small-group discussion, e-learning, and clinical skills sessions. These courses include subject areas such as biochemistry, cardiology, neurology, and community health. They average around three weeks in length with a range of two to six weeks depending on the topic. Course activities typically occupy four to six hours of instructional time daily during these periods. The final two years are referred to as “clerkship” and involve six to twelve week rotations core rotations in disciplines such as family medicine, internal medicine, and general surgery that are delivered at either urban or rural teaching sites. Two to four week elective rotations may be taken at external sites.

The Program Evaluation Subcommittee (PESC) at Memorial University of Newfoundland is the internal curriculum evaluation oversight committee for the undergraduate medical program. PESC examines a variety of data including student feedback surveys, Canadian licensing exam results, residency match reports, standardized exam results (e.g., National Board of Medical Examiners), student exit interviews at graduation, medical graduate surveys, and faculty opinion in a holistic assessment of the medical curriculum. It is also evaluated externally at regular intervals by an accreditation process. This is conducted by the joint Canadian/American body known as the Committee on the Accreditation of Canadian Medical Schools/Liaison Committee on Medical Education.

### Objective

The primary objective of our study is to describe the “intensive course review” protocol that is triggered by student course evaluation feedback. Our secondary objective is to report a retrospective analysis of the effectiveness of this intensive course review in improving overall course ratings (defined by number of courses exceeding an overall rating >3.5/5).

### Intensive course review protocol

**Fig. 1:**
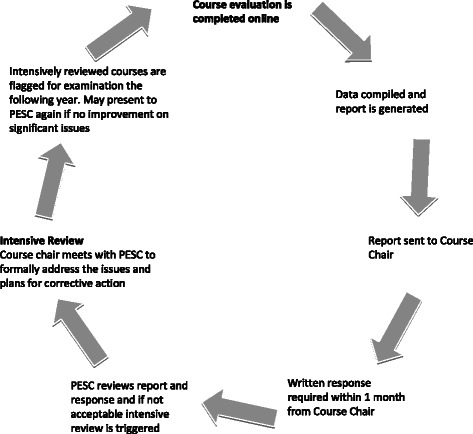
Outline of the intensive course review protocol. Course evaluation questionnaires are completed by students and a report is then complied by the Program Evaluation Subcommittee. Reports are then sent to Course Chairs for courses not meeting the minimal benchmark with a written response is required within 1 month. Based on the response, an intensive course review may be initiated requiring a detailed written action plan addressing all course deficiencies and in-person meeting with the course chair. Courses undergoing intensive review and flagged for reassessment the following year

The intensive course review protocol for examining student course evaluations is illustrated in Fig. 1. We circulate course evaluation questionnaires electronically to students after completion of a course. Students evaluate their courses on a variety of dimensions using a 5-point Likert scale (see Additional file 1).

Those courses not meeting established standards undergo an “intensive course review”. Three criteria determine when an intensive review of a course is necessary. These include: 1) an overall mean rating below 3.5/5.0; 2) a decrease in overall mean course rating of >0.5/5.0 over one year; or 3) a committee-identified critical course issue (e.g., chronic recurring issues or poor standardized exam results in courses otherwise meeting the benchmark). The <3.5 threshold and the decrease of 0.5 was established based upon the observation of marked increases in negative comments with these course ratings below this level.

When undergoing an intensive review, the course chair presents an action plan in person to PESC addressing course deficiencies and detailing steps to resolve identified problems. This provides an avenue for course chair, faculty, and student discussion of the proposed action plan. Student liaisons provide input to the committee on strategies for course improvement. Results of medical licensing exams and standardized exams are considered. Courses requiring an intensive review are flagged for reassessment to track the impact of any implemented changes. PESC may require a course chair to meet with the committee again if critical issues remain or if course ratings do not improve. In courses not requiring intensive review, both positive and negative feedback is communicated to course chairs to aid in ongoing curriculum enhancement. This model for student evaluation of curriculum was implemented during the 2006/07 academic year.

## Methods

Ethics approval is not required for secondary use of non-identifiable data or program evaluation at our institution as per the local Health Research Ethics Authority [11] and Canadian Tri-Council Policy Statement guidelines (articles 2.4-2.5) [12]. Completion of the questionnaire was considered implied consent to provide course evaluation feedback. All responses were anonymous and no identifiable information was collected. We examined student ratings for pre-clerkship courses from 2006 to 2011. Course surveys were distributed on paper in the 2006/07 and 2007/08 years during the final lecture of a course. Starting in the 2008/09 year they were released via email and completed online after a course was finished. The content of the surveys remained identical. In addition, medical school admissions criteria, course chairs, and course instructors remained consistent during the study period. Most of the courses were unchanged, although a small number of courses were discontinued or restructured early in the study period. Such courses with incomplete data were excluded from the analysis. We excluded feedback data from clerkship rotations since none required an intensive course review during our study period. The proportion of courses below the 3.5 benchmark and their median ratings were calculated from 2006 to 2011.

To examine the differences in the course ratings across academic years, a non-parametric analysis using Kruskall-Wallis test (SPSS V21) was conducted with alpha set at 0.05. Post-hoc tests were conducted using Mann-Whitney U tests with the Bonferroni correction (alpha set at 0.01). Response rates for student evaluations of each course included in the analysis were also calculated.

## Results

**Fig. 2:**
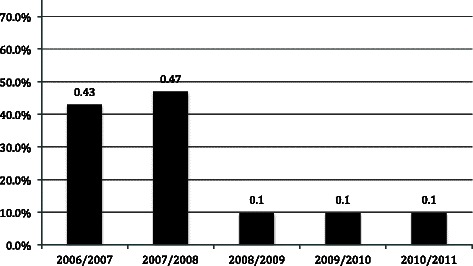
Percentage of courses below benchmark by academic year. Courses below the minimum benchmark of a 3.5/5 rating from 2006 to 2011. The intensive course review protocol was initiated in the 2006/07 year. A Kruskall-Wallis test revealed statistically significant differences across all five academic years (p < 0.001)

We included 30 independent units of coursework per academic year in our analysis (total sample n = 150). We excluded 4 courses due to missing data – none of which required an intensive review. In the 2006/2007 academic year, 13/30 courses (43.3 %) did not meet the minimum benchmark and were placed under intensive review. By 2010/2011, only 3/30 courses (10.0 %) were still below the minimum benchmark (Fig. 2). Median course ratings have trended upward since implementing our protocol (Table 1). The only criterion utilized to trigger an intensive course review during our study period was our <3.5 benchmark. The 3 courses that remained below the benchmark were consistent during follow-up.

**Table 1:** Median ratings for courses by academic year and mann whitney u tests comparing academic years to the pre-intensive course review period^a^

Academic Year	Enrolled Pre-clerkship students^c^	Median rating (interquartile range)	P-value	Effect size (η^2^)	U score	Z score	R score
2006/07^b^	120	3.50 (3.20, 4.00)	-	-	-	-	-
2007/08	120	3.50 (3.30, 3.70)	0.614	NS	NS	NS	NS
2008/09	124	4.10 (3.80, 4.30)	<0.001	0.222	200.0	−3.705	0.676
2009/10	128	4.15 (3.70, 4.30)	0.001	0.246	221.5	−3.386	0.618
2010/11	128	4.00 (3.80, 4.20)	0.004	0.283	255.0	−2.890	0.528

^a^Mann Whitney U, compared to 2006/07

^b^Pre-Intensive Course Review Protocol

^c^Based on enrollment data [15]

A Kruskall-Wallis test revealed statistically significant differences in course ratings across five academic years (2006/07, n = 30, 2007/08, n = 30, 2008/09, n = 30, 2009/10, n = 30, 2010/11, n = 30), ×^2^ = 31.798, n = 150, degrees of freedom = 4, p < 0.001. Mann-Whitney U Tests revealed that course ratings, compared to 2006/07, were significantly higher in 2008/09, 2009/10, and 2010/11 (Table 1).

Since the 2006/07 academic year, there has been a steady decline in student response rates. In 2006/07 the response rate was 76.5 % and this decreased to 49.7 % in 2010/11.

## Discussion

There is little practical information in the literature on how medical schools respond to poor course ratings or the impact of any intervention. We describe the intensive course review protocol used at Memorial University of Newfoundland in St. John’s, Canada. Our analysis suggests that implementing an intensive course review protocol can have a substantial and significant influence on improving pre-clerkship student course evaluation in an undergraduate medical program.

There are several components of our approach that may account for these results. The intensive course review protocol is characterized by a systematic process for responding to negative student evaluations using pre-determined benchmarks for comprehensive course review. The pre-clerkship and clerkship student representatives are full voting members of the committee and provide a valuable liaison between the PESC and students. This ensures that students are informed about the impact of their evaluations on courses. They can also provide input directly to the committee on student concerns and suggestions for improvement. The PESC committee centralizes monitoring of course ratings and provides evaluation data to course chairs. This gives student course evaluation a higher profile within the administrative structure of the medical school. But perhaps most importantly, the intensive course review protocol has a high level of accountability built in for course chairs through the requirement to develop and present a formal action plan. It also allows PESC to consider course chair opinion when assessing course evaluation feedback. Together, these factors help to ensure a transparent and effective process for managing negative course evaluations.

Improvement in course ratings may be due to a number of factors. We believe changes in the course content and delivery resulting from intensive course reviews likely plays a substantial role in improved student ratings. The intensive course review also typically includes improved communication on course objectives and structure that may improve student ratings. The “cohort effect” whereby students in one academic year may over or undervalue certain aspects of the teaching style and curriculum compared to prior years may explain some of our results. However, the admissions policies, curriculum structure, course objectives and key faculty were consistent during our study period. As well, the improvements in student course evaluations were maintained over three consecutive cohorts following two consecutive cohorts with lower median ratings making it less likely that cohort effects are a significant factor in the improved scores. In addition, the timing of the improvements is consistent with the introduction of the intensive course review protocol suggesting that it is having an impact.

There remain a number of challenges in implementing the course evaluation process. The most critical is maintaining acceptable response rates. There is conflicting evidence concerning the use of electronic surveys for course evaluations and whether they increase or decrease response rates [13]. However, since the implementation of electronic course evaluations by PESC, response rates for the pre-clerkship courses have steadily dropped from 74.8 % in 2006/07 to 49.7 % in 2010/11. When student participation falls below 50 %, there is a real threat to the validity and reliability of the evaluations that may result in faculty disregarding them [14]. Improved course ratings could be due to bias introduced by lower response rates. However, it is possible that students who dissatisfied with courses tend to respond to surveys and therefore results underestimate improvements. PESC is currently exploring options to increase response rates such as incentive programs or a return to paper evaluations.

There are several limitations to this descriptive study. Firstly, it relies primarily on survey data from students that may not accurately capture all curriculum deficiencies. We only examine the effect on pre-clerkship courses since no clerkship rotations required an intensive review during our study period limiting the generalizability of our findings to upper years. Another limitation is that outcome-based measures are not directly used to trigger an intensive course review. However, PESC does consider results of Canadian medical licensing exams and other objective measures when examining courses undergoing an intensive review. There is also the risk of regression to the mean given the low values of many courses at baseline. We did take repeated measures of all of the courses over time with consistent improvement that suggests our data is valid. As well, visual inspection of individual course data reveals that the median course ratings for most courses remained the same or increased over the study period suggesting that regression to the mean is not having a large impact. Unfortunately, we did not have access to within-subject data and therefore cannot complete an analysis of covariance to rule out regression to the mean bias.

## Conclusion

Our analysis demonstrates that the intensive course review protocol has significantly and meaningfully (η^2^: 0.222-0.283) improved pre-clerkship course ratings at our institution. Our success is likely related to having a transparent and systematic process to address course feedback with input from multiple stakeholders. Our results may be not generalizable beyond pre-clerkship courses because we were unable to examine clinical rotations since none required a review. Declining student response rates represent an ongoing challenge to interpreting data.

## Additional file

Additional file 1:
**Sample Student Feedback Survey.**

## References

[1] Hendry GD, Cumming RG, Lyon PM, Gordon J (2007). Student-centred course evaluation in a four-year, problem based medical programme: issues in collection and management of feedback. Assess Eval High Educ.

[2] Krantz Girod C, Raphael Bonvin R, Lanares J, Cuenot S, Feihl F, Bosman F, Waeber B (2004). Stability of repeated student evaluations of teaching in the second preclinical year of a medical curriculum. Assess Eval High Educ.

[3] Snell L, Tallett S, Haist S, Hays R, Norcini J, Prince K (2000). A review of the evaluation of clinical teaching: new perspectives and challenges. Med Educ.

[4] Abrahams MB, Friedman CP (1996). Preclinical course-evaluation methods at U.S. and Canadian medical schools. Acad Med.

[5] Kogan JR, Shea JA (2007). Course evaluation in medical education. Teaching Teacher Educ.

[6] Aleamoni LM (1999). Student rating myths versus research facts from 1924 to 1998. J Person Eval Educ.

[7] Copeland HL, Hewson MG (2000). Developing and testing an instrument to measure the effectiveness of clinical teaching in an academic medical center. Acad Med.

[8] Rudland JR, Pippard MJ, Rennie SC (2005). Comparison of opinions and profiles of late or non-responding medical students with initial responders to a course evaluation questionnaire. Med Teach.

[9] Gibson KA, Boyle P, Black DA, Cunningham M, Grimm MC, McNeil HP (2008). Enhancing evaluation in an undergraduate medical education program. Acad Med.

[10] Watson S (2003). Closing the feedback loop: ensuring effective action from student feedback. Tert Educ Manag.

[11] Health Research Ethics Authority. Ethics Review Required? May 6, 2015. Available at http://www.hrea.ca/Ethics-Review-Required.aspx. Accessed May 6, 2015.

[12] Canadian Institutes of Health Research, Natural Sciences and Engineering Research Council of Canada, and Social Sciences and Humanities Research Council of Canada, Tri-Council Policy Statement: Ethical Conduct for Research Involving Humans, December 2010. Available at: http://www.pre.ethics.gc.ca/pdf/eng/tcps2/TCPS_2_FINAL_Web.pdf. Accessed May 6, 2015.

[13] Moss J, Hendry G (2002). Use of electronic surveys in course evalution. Br J Educ Technol.

[14] Gravestock P & Gregor-Greenleaf E. Student Course Evaluations: Research, Models and Trends Report. The Higher Education Quality Council of Ontario: 2008 Accessed 10/1/2012, 2012 from http://www.heqco.ca/SiteCollectionDocuments/Student%20Course%20Evaluations.pdf.

[15] Canadian Medical Education Statistics. The Association of Faculties of Medicine of Canada. 2014. Accessed December 28, 2014 from https://www.afmc.ca/pdf/CMES2014-Complete-Optimized.pdf.

