# Supplementary material for: Making medical student course evaluations meaningful: implementation of an intensive course review protocol
Source: BMC Med Educ. 2015 Jun 4;15:99. doi: 10.1186/s12909-015-0387-1 (PMC4460774; doi:10.1186/s12909-015-0387-1)
Supplement: Additional file 1: — Sample Student Feedback Survey. [file 12909_2015_387_MOESM1_ESM.doc]

# Appendix 1 – Sample Student Feedback Survey

# Memorial University Medical School Pre-Clerkship Course evaluation

**COURSE NAME**

Your input is very important. Your responses are collated and distributed to the course chair, pre-clerkship chair, undergraduate dean, chair of the undergraduate studies committee and program evaluation committee. These reports are considered and discussed by the relevant committees with several goals as follows:

- Problems with respect to course content, organization, effectiveness etc. can be identified and initiatives undertaken to rectify deficiencies
- New effective initiatives undertaken in a course can be identified and promoted for inclusion in other courses
- Faculty can use these reports to enhance the strengths you have identified or correct the weaknesses you have described. Some faculty may use this documentation as part of their promotions applications.

**Section A: Regarding the course as a whole**

***Indicate your level of agreement with each statement by choosing the appropriate number.***

*Key: 1= disagree strongly 2=disagree 3=agree 4=agree strongly 5=agree very strongly*

| ***Statements related to the course as a whole*** | |
| --- | --- |
| 1. The learning objectives of the course were clear. | **1 2 3 4 5** |
| 1. Lectures and other course activities were related to the objectives. | **1 2 3 4 5** |
| 1. The organization of the course with respect to its design and structure helped my learning. | **1 2 3 4 5** |
| 1. The overall workload for this course was about right. | **1 2 3 4 5** |
| 1. I gained a good understanding of the concepts and principles. | **1 2 3 4 5** |
| 1. I increased my interest in the subject matter of this course. | **1 2 3 4 5** |
| 1. I understood the clinical relevance of this course. | **1 2 3 4 5** |
| 1. On the whole, this was a good course. | **1 2 3 4 5** |

***Indicate your level of agreement with each statement by choosing the appropriate number.***

*Key: 1= disagree strongly 2=disagree 3=agree 4=agree strongly 5=agree very strongly*

| ***Statements related to online course resources*** |  |
| --- | --- |
| 1. The design and structure of the online materials were reflective of the course. | **1 2 3 4 5** |
| 1. It was easy to navigate the online course site (WebCT). | **1 2 3 4 5** |
| 1. The organization of the course with respect to its implementation was effective (the course ran as it should, teaching materials were available as promised etc.) | **1 2 3 4 5** |
| 1. Materials posted online (WebCT) adequately represented what was covered in the course. | **1 2 3 4 5** |
| 1. Information and resources posted online (WebCT) were accurate. | **1 2 3 4 5** |
| 1. Online course resources were available when I needed them. | **1 2 3 4 5** |
| 1. The technical set up (computer access, speed of connections, size of files, etc.) allowed for good access to online course information and materials. | **1 2 3 4 5** |

***Comments on the course as a whole:***

Please make general comments about the course as a whole, considering the following elements:

- Objectives
- Organization
- Redundancies with other courses
- Key strengths and/or weaknesses
- Omissions/Deficiencies
- Overall impression

|  |
| --- |

***Section B: Faculty evaluation***

Please rate the overall effectiveness of individual faculty to facilitate your learning. In the comments sections please make selected comments regarding strengths and weakness of individual faculty. **Comments should be reasoned judgments and objective observations. Both positive and negative comments are encouraged. Inappropriate and unprofessional derogatory comments will be disregarded/deleted and may result in the complete evaluation form being discarded without being further considered.** All other comments will be forwarded to individual faculty. You may consider some or all of the following categories in your comments:

- Style
- Organization
- Content
- Effective use of time (too much/too little material)
- Student interaction (responds to questions, stimulates questions)
- Effective use of AV materials and appropriate application
- Stimulated to learn more
- Suggestions for improvements

***Indicate your rating of the individual’s teaching by choosing the appropriate number.***

***Key: 1=highly ineffective; 2= notable weakness; 3=fair; 4= good; 5=highly effective***

| ***Faculty name/topics taught*** | ***Rating*** |
| --- | --- |
|  | **1 2 3 4 5** |
|  | **Comments:** |
|  | **1 2 3 4 5** |
|  | **Comments:** |

***Section C: Review of selected objectives & subcomponents of the course:***

Please consider both the topic as a whole (effectiveness of teaching of this objective, aspects over-included/ omitted, etc.) and the quality/effectiveness of individual teaching formats used (laboratory or tutorial vs. lecture). **Remember, this section addresses the overall teaching of a topic and not of an individual professor.**

***Indicate your rating of the topic by choosing the appropriate number.***

***Key: 1=highly ineffective; 2= notable weakness; 3=fair; 4= good; 5=highly effective***

| ***Subject*** | ***Rating*** |
| --- | --- |
|  | **1 2 3 4 5** |
|  | **Comments:** |
|  | **1 2 3 4 5** |
|  | **Comments:** |

***Section D: Evaluation of the text book***

| ***Statement regarding text(s):*** | **Rating and comments:**  **1= strongly disagree; 2=disagree; 3=neutral/mixed opinion;**  **4=agree; 5 =strongly agree** |
| --- | --- |
| The recommended text was a useful aid to my learning. | ***1 2 3 4 5*** |
|  | ***Comments:*** |

***Section E: Evaluation of the examination***

| ***Statement regarding exam (s):*** | ***Rating and comments:***  ***1= strongly disagree; 2=disagree; 3=neutral/mixed opinion;***  ***4=agree; 5 =strongly agree*** |
| --- | --- |
| The exam(s) accurately reflected the course objectives. | **1 2 3 4 5** |
|  | **Comments:** |
| The exam(s) accurately reflected the course content. | **1 2 3 4 5** |
|  | **Comments:** |

**Thank you for your feedback!**
